# New Insight on the Sublethal Effect of Bt-Cry1Ab in *Spodoptera litura* (Fabricius): Tissular Distribution of Cry1Ab, Ultrastructural Alterations and the Lysosomal Response

**DOI:** 10.3390/insects16010010

**Published:** 2024-12-27

**Authors:** Yan-Jue Wang, Ya-Qin Shen, Ying-Dan Xiao, Xue Yang, Shao-Peng Hao, Jian-Qiu Liu, Xiao-Xue Yang, Kazuei Mita, Ya-Jing Xu

**Affiliations:** 1 Integrative Science Center of Germplasm Creation in Western China (Chongqing) Science City, Biological Science Research Center, Southwest University, Chongqing 400715, China; 2 Department of Neurosurgery and Glioma Medical Research Center, Southwest Hospital, Third Military Medical University, Chongqing 400038, China

**Keywords:** *S. litura*, Bt-Cry1Ab, sublethal effect, tissular distribution, TEM, lysosome

## Abstract

Bt has been applied as a gene source for insect-resistant transgenic crops, which represents efficient control of insect pests. In this study, we evaluated the pesticidal specificity of one Bt maize strain, DBN9936, that expresses Cry1Ab protein in *Spodoptera litura* larvae. The results showed that this Bt maize is active against the younger larvae while causing a sublethal effect on older larvae. To further assess the biological responses of *S. litura* under sublethal Cry1Ab exposure, the relative concentrations of Cry1Ab in different tissues of fifth instar larvae were investigated, indicating that Cry1Ab is transported along the gut and out of the body via excretion or into the hemocoel and lead to sequestration by molting. Furthermore, the result of ultrastructural observation in sublethal Cry1Ab-treated midgut, namely an increase in lysosome number, with the lysosomal activity activation simultaneously provides a strong indication that lysosome plays an active role in response to sublethal Cry1Ab exposure.

## Simple Summary

Nowadays, genetically modified Bt crops or Bt spray products are the main approaches used in agriculture management and are active against agricultural pests. *Spodoptera litura* is a highly polyphagous pest that causes damage to various crops. In this study, we evaluated the pesticidal specificity of one Bt maize strain, DBN9936, that expresses Cry1Ab protein to *S. litura* larvae. The results showed that this Bt maize caused a sublethal effect on older larvae. To further assess the biological responses of *S. litura* under sublethal Cry1Ab exposure, the relative concentrations of Cry1Ab in different tissues of fifth-instar larvae were investigated. Furthermore, ultrastructural observation and the lysosomal activity detection in sublethal Cry1Ab-treated midgut provide a strong indication that lysosome plays an active role in response to sublethal Cry1Ab exposure.

## 1. Introduction

*Bacillus thuringiensis* (Bt) has developed into a worldwide biological control agent because of its high specificity to target insects, its safety for non-target organisms such as humans, vertebrates, and plants, and its excellent biodegradability [1,2]. The genetic modification of Bt crops is one of the important approaches used in agricultural pest management at present time and is effective against pests in the orders Lepidoptera and Coleoptera [3,4]. Since the approval of commercialized GM Bt crops in the United States, their adoption and the area of land on which they are cultivated have steadily increased [5]. The three-domain Cry (3d-Cry) toxins produced by Bt represent the most exploited group of biopesticides. In China, first-generation Bt transgenic cotton expressing Cry1Ac protein began to be commercially planted in 1997 to combat the cotton bollworm *Helicoverpa armigera* (Hübner) [6,7]. Although Bt crops are considered safe for human health and the environment, only GM Bt cotton has been widely cultivated in China thus far. However, several Bt transgenic events have sequentially received biosafety certificates, including two Bt rice lines (Huahui-1, expressing BtCry1Ac/BtCry1Ab, and Xianyou-63, expressing BtCry1Ab/Ac) and Bt maize lines (Ruifeng125, expressing BtCry1Ab/BtCry2Aj and DBN9936, expressing BtCry1Ab, among others) [8,9,10]. According to the roadmap of “non-edible crops, indirectly edible crops, and directly edible crops” for the development of GM technology, it is reasonable to believe that China will soon enter an era of commercially cultivating GM Bt maize for feed and feed processing [11]. However, the large-scale and long-term commercial planting of Bt cotton has resulted in field-evolved resistance in *H. armigera* in China [12]. Subsequently, practical resistance to the Cry toxin has been documented for several pest species, such as *Spodoptera frugiperda* (Smith) [13], *Pectinophora gossypiella* (Saunders) [14,15], and *Plutella xylostella* (L.) [16] in the Bt crop.

Numerous studies have highlighted that sublethal effects from Bt exposure in the field may contribute to the development of Bt resistance, affecting the life history traits and critical physiological functions of target insects [17,18]. Experiments estimating the sublethal effects of Bt on agricultural pests have reported developmental polymorphism in *Choristoneura fumiferana* (Clemens), high fitness costs in the biology and development of *H. armigera*, and reductions in larval weight with increased predation in *Helicoverpa zea* (Boddie) [19,20,21]. Additionally, expression changes in detoxification, immune response, energy metabolism, and resistance-related genes in response to a sublethal dose of Bt toxins have elucidated the molecular basis of Bt’s sublethal effect [22,23,24,25]. Despite these findings, more studies are needed to evaluate the Bt’s sublethal effects on larvae to help elucidate Bt’s resistance mechanism and integrate Bt application in the farmland.

*Spodoptera litura* (Fabricius) is a highly polyphagous pest that readily infects young plants and feeds on tender leaves, which more easily causes complete yield losses because of the break of growing points [26]. In a previous study, we found that *S. litura* larvae exposed to sublethal doses of Cry1Ab toxin exhibited apparent histomorphological and proteotranscriptomic alterations in the midgut that are associated with the risk of pest resistance development [27]. Further studies on *S. litura*’s response to sublethal Cry1Ab exposure would increase our understanding of the Bt’s sublethal effect. In this study, we measured the mortality of different *S. litura* instar larvae feeding on DBN9936 Bt maize event to assess the control efficacy in Bt maize fields and examined the fine structure and properties of Cry1Ab-treated midgut samples using a transmission electron microscope (TEM). We also reported, for the first time, the content of Cry1Ab toxin within the different tissues of fifth instar *S. litura* larvae after sublethal Cry1Ab exposure and explored changes in lysosomal activity. The purpose of this present study was to investigate the sublethal effects of Cry1Ab on cytopathological changes and the responsive molecular mechanism of *S. litura* larvae.

## 2. Materials and Methods

### 2.1. Insect Rearing and Bioassay of S. litura Larvae on Transgenic Cry1Ab Maize

The *S. litura* strain was originally collected from a field in Xin Yang, He Nan, China. We purchased the insects from Ke Yun Biology in June 2020 and kept them on an artificial diet at 27 ± 2 °C, with a 14:10 h (light:dark) photoperiod and 60–70% relative humidity at the biological science research center of Southwest University (Chongqing, China). Transgenic maize (*Zea mays* L.) plants (event DBN9936; Da Bei Nong group: DBN9936) expressing Cry1Ab protein and the common maize variety (control) were used in this study. Both maize varieties were sown in a non-irrigated plot by applying compound fertilizer, and they were kept in a greenhouse at Southwest University. No pesticides were applied during the growing period. The 1st–5th instar larvae of *S. litura* were reared on cleaned and dried maize leaves after molting within 6 h, respectively. A bioassay was conducted with three replicates, and 10 insects were tested for each replicate. Fresh maize leaves at stages V1–V3 were collected from the control and transgenic plots and placed in 125 mL plastic cups for feed. Larval mortality or the development was recorded daily for 120 h. Mortality curves of different instar larvae were generated based on the mortality rates and feeding time.

### 2.2. Enzyme-Linked Immunosorbent Assay (ELISA)

To standardize the developmental stage of the experimental larvae, the 5th instar larvae were obtained for further assay when molting within 6 h. To further elucidate the Cry1Ab cytotoxicity in the whole bodies of the larvae under sublethal exposure, we used an ELISA to investigate the distribution of Cry1Ab in some major tissues, including the foregut, midgut, hindgut, Malpighian tubules, fat body, hemolymph, feces, cuticle, and saliva. We first detected the Cry1Ab contents in the leaf samples from two maize varieties, namely Bt-Cry1Ab proteins from the ELISA kit and Cry1Ab protein, which is used for diet supplementation, respectively, to ensure the accuracy of Cry1Ab detection. Different tissues from 5th instar larvae fed with DBN9936 maize leaves for 72 h were obtained and used as Cry1Ab exposure samples, and samples from larvae fed common maize leaves served as the control group. Additionally, the caste skin and new cuticle were obtained when the larvae formed an epidermis. Furthermore, we determined the Cry1Ab contents on 5th instar larvae of *S. litura* that were fed an artificial diet supplemented with a sublethal concentration of Cry1Ab (Meiyan Agricultural Technology Co., Ltd., Beijing, China), namely 140 μg/g, or larvae fed an artificial diet without Cry1Ab for further confirmation of the Cry1Ab distribution and concentration in different tissues. Each treatment was prepared with three replications.

Phosphate-buffer solution with Tween-20 (PBST; 8 mM Na_2_-HPO_4_, 2 mM KH_2_PO_4_, 10 mM KCl, 140 mM NaCl, 0.05% Tween-20, and pH 7.4) was added to the samples at a sample (g):PBST (mL) ratio of 1:10, and then the samples were fully ground using a Tissuelyser (Tiangen, Beijing, China). Following centrifugation at 12,000 rpm for 10 min, the supernatants were analyzed using a BtCry1Ab/1Ac ELISA kit (Agdia, Elkhart, IN, USA). According to the manufacturer’s instructions, the test and control samples were added to the ELISA plate (100 µL/well) and incubated for 2 h at 37 °C in an incubation shaker (Bio shark, Saitama, Japan). The unbound proteins were removed via seven washes with PBST, and the ELISA plate was developed with a tetramethylbenzidine (TMB) substrate solution. The absorbances were measured at 650 nm with a microplate reader (Synergy H4, Biotek, Winooski, VT, USA).

### 2.3. Transmission Electron Microscopy (TEM)

TEM was used to view the ultrastructure changes in the midgut of *S. litura* after 5th instar larvae were exposed to a sublethal level of Cry1Ab for 48 h. The specimens were fixed for 3 h in 3% glutaraldehyde (Lilai, Chengdu, China) overnight at 4 °C; then, they were repeatedly rinsed in phosphate buffer at pH 7.4 thrice and post-fixed for 2 h at 4 °C in 1% osmium tetroxide (Lilai, Chengdu, China). The samples were then repeatedly rinsed in the same buffer and dehydrated with ascending ethanol concentrations (30–100%) for 15 min at each concentration and 100% acetone three times. Finally, the samples were embedded in an Araldite-Epon (Embed-812) mixture. Afterward, ultrathin sections around the midgut of approximately 70 nm around the midgut were cut with a Leica EMUC7 ultramicrotome, collected on a 200-mesh copper grid, and stained with uranyl acetate for 15 min and lead citrate for 2 min. The samples were examined using JEM 1400 FLASH TEM (JEOL Ltd., Tokyo, Japan), and digital images were obtained.

### 2.4. Acid Phosphatase (ACP) Enzymatic Activity Assay of the Cry1Ab-Treated Midgut

The activity of ACP, a marker enzyme for lysosomes, was used to quantify the activity of lysosomes. We measured the enzyme activity of ACP using activity assay kits (Beyotime, Shanghai, China) in 96-well microplates in a Synergy H4 microplate reader (BioTek, Winooski, VT, USA). After the 5th instar larvae were exposed to a sublethal level of 140 μg/g sublethal level of Cry1Ab for 48 h, the cell lysis buffer without inhibitors (Beyotime, Shanghai, China) was added to the samples at a sample (g):lysing solution (mL) ratio of 1:10, and the midgut supernatant was used to obtain the absorbance data. According to the activity assay kit instructions, the absorbance data of para-nitrophenol was used to calculate the enzymatic activity of acid phosphatase. Each assay was performed in triplicate and averaged for each biological replicate.

### 2.5. RNA Extraction and Quantitative Real-Time Reverse Transcription PCR (qPCR) Analysis

V-ATPases are proton pumps that are required for the acidification of the lysosomes and other membrane-bound compartments, so the expression of vacuolar-type ATPase (V-ATPase) genes was determined after the 5th instar larvae were exposed to a 140 μg/g dose of Cry1Ab for 48h using qPCR. Three biological replicates of the larvae midguts were collected for RNA extraction using the Trizol reagent (Invitrogen, Carlsbad, CA, USA) according to the manufacturer’s protocol. Hifair^®^ III 1st Strand cDNA Synthesis (Yeason, Shanghai, China) was used to remove any contaminating genomic DNA and synthesize cDNA from 1 μg of the total RNA. The real-time quantitative PCR (qPCR) assays used Hieff^®^ qPCR SYBR Green system (Yeason) based on the manufacturer’s instructions. All the specific primers used in the qPCR are listed in Table 1. We used ribosomal protein L7A (RPL7A) as the reference genes according to a previous study [28]. The expression levels were calculated using the 2^−ΔΔCt^ comparative CT method.

### 2.6. Red Lysotracker Staining of Cells

LysoTracker Red DND-99 generally aggregates on the spherical organelles and is suitable for observing the functional changes in lysosomes. The BmE (embryo cell line of *Bombyx mori*) cells were seeded at a density of 1 × 10^5^ cells per well in 24-well plates and maintained at a 28 °C incubator in Grace’s medium (Thermo Fisher Scientific, Waltham, MA, USA) supplemented with 10% (*V*/*V*) fetal bovine serum (FBS) (Thermo Fisher Scientific, Waltham, MA, USA). The next day, fresh media containing 0.1 M or 0.2 M Cry1Ab toxin replaced old media at 0 h, 12 h, and 24 h, respectively. After 24 h, all cells were stained with 50 nm LysoTracker Red DND-99 (Yeasen, Shanghai, China) with Grace’s medium for 30 min in the dark at room temperature. The lysotracker cells were viewed under a fluorescent microscope (BX53, Olympus, Tokyo, Japan).

### 2.7. Data Analysis

The data are represented as the triplicate mean ± SD (standard deviation). Between-group differences were compared with independent Student’s *t*-tests, with *p* ≤ 0.05 considered statistically significant. Statistical significance was determined using SPSS 20.0 software (IBM, Armonk, NY, USA). All data were visualized using Origin version 9.1 (OriginLab, Northampton, MA, USA).

## 3. Results

### 3.1. S. litura Bioassay

**Figure 1:**
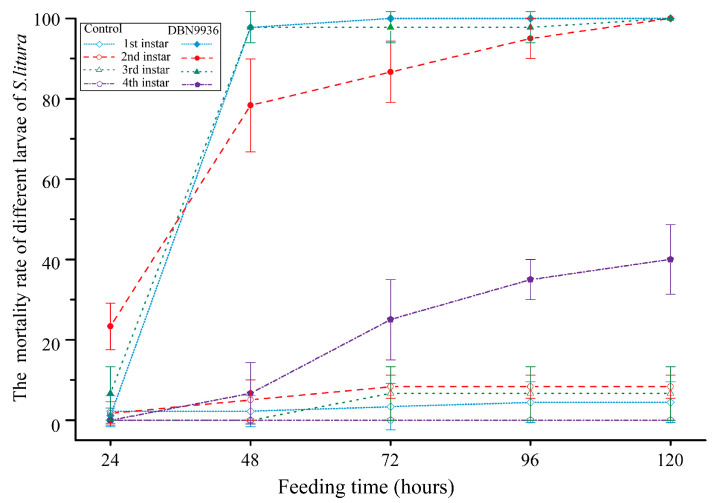
The mortality rate of *S. litura* feed on Bt maize leaf. Data show that DBN9936 maize feeds first–fourth instar larvae for 72 h. Scatter heights and error bars showed means ± SD.

*S. litura* bioassays of younger larvae first–third instar larvae on DBN9936 maize leaves showed almost 100% mortality after 96 h of feeding compared with the mortality rate in fourth instar larvae, which decreased to about 40%. Contrarily, the survival of *S. litura* larvae that were fed control maize leaves was marginally impacted (Figure 1). Furthermore, when the DBN9936 maize was fed to the fifth instar larvae, the development duration was delayed from 2.34 ± 0.23 d to 4.06 ± 0.40 d.

### 3.2. ELISA Analysis of Cry1Ab Protein Content in Cry1Ab Feeding Larvae

**Figure 2:**
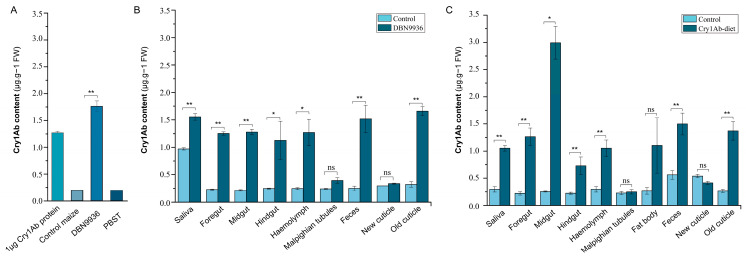
Mean expression levels of Cry1Ab in different tissues of *S.litura* larvae after 72 h Cry1Ab feeding. (**A**) The control samples and (**B**) different tissues from larvae feeding on DBN9936 and (**C**) feeding on Cry1Ab-diet. The asterisk on the bars indicates significantly different means (*: 0.01 ≤ *p* ≤ 0.05; **: *p* ≤ 0.01; ns: no significance) performed by *t*-test.

Cry1Ab protein was readily detectable in the sample that extracted proteins from DBN9936 leaves when the activated Cry1Ab protein was used as the positive control but was not detected in the control maize leaves, where PBST as the blank control (Figure 2A). Compared to the tissues obtained from larvae feeding on the control maize leaves, the Cry1Ab content was significantly distributed in the saliva, foregut, midgut, hindgut, hemolymph, feces, and old cuticles of the larvae fed with the DBN9936 maize leaves for 72 h, but Cry1Ab was not detectable in Malpighian tubules or the newly developed cuticles. Cry1Ab failed to accumulate in the fat bodies of the larvae that fed on both types of maize leaves (Figure 2B). We used bioassays to further investigate Cry1Ab accumulation in specific tissues after feeding the larvae an artificial diet with a sublethal concentration of Cry1Ab that inhibits larvae weight without causing mortality for 72 h. The Cry1Ab contents in different tissues of *S. litura* larvae after feeding with an artificial diet containing a sublethal dose of Cry1Ab presented the same tendency as stated above; nevertheless, there was no significant difference in the concentration of Cry1Ab in fat bodies (Figure 2C).

### 3.3. Ultrastructural Changes in the Midgut of S. litura Larvae After Cry1Ab Exposure

**Figure 3:**
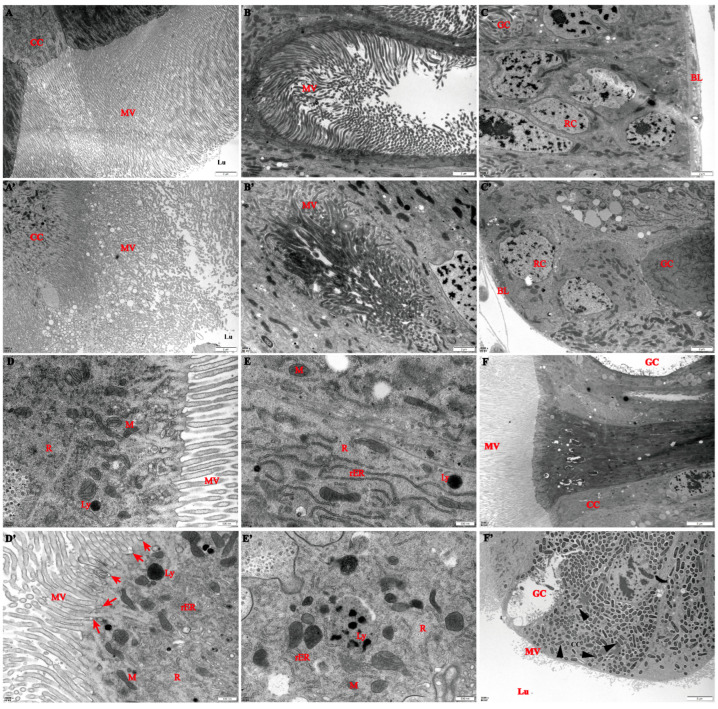
Ultrastructural changes in the midgut of *S. litura* fifth instar larvae after treatment with a sublethal concentration of Cry1Ab for 48 h. (**A**–**F**) The control group and (**A’**–**F’**) the Cry1Ab treatment group. Columnar cell (CC), Microvilli (MV), Midgut lumen (Lu), Goblet cells (GC), Regenerative cells (RC), Basal lamina (BL), Ribosome (R), Mitochondria (M), Lysosomes (Ly), Rough endoplasmic reticulum (rER), shallow infoldings on apical membrane surface (arrows), Rod-shaped bacteria (triangular arrowheads).

After being exposed to a sublethal dose of Cry1Ab for 48 h treatment, the ultrastructure of the midguts in fifth instar *S. litura* larvae revealed substantial ultrastructural differences based on a TEM observation. The typical structure of the control larvae midgut was found to be typical, showing that each epithelial cell was filled with numerous and regularly arranged microvilli, the basal lamina possessed an evident border (Figure 3A–C), and the organelles, such as the ribosome, mitochondria, endoplasmic reticulum, and lysosome, were complete (Figure 3D–F). After Cry1Ab exposure, the midgut exhibited significant ultrastructural changes with shedding, fragmented, and destroyed microvilli, a reduction in the number of regeneration cells, and a breakdown of the basal lamina (Figure 3A’–C’), which was also discovered in our previous study through hematoxylin–eosin Staining [27]. Additionally, some condensed mitochondria with a loss of cristae were present after exposure to a sublethal Cry1Ab dose. A fragmented, rough endoplasmic reticulum (rER) was also observed throughout the cytoplasm, and ribosomes were shed from the rER membrane. Meanwhile, increased quantities of lysosomes scattered in the cytoplasm and the shallow infoldings on the apical cell surface were observed (Figure 3D’–E’). Another predominant finding was that some severely destroyed column cells were packed with rod-shaped bacteria (Figure 3F’).

### 3.4. Lysosome Activity and Localization

**Figure 4:**
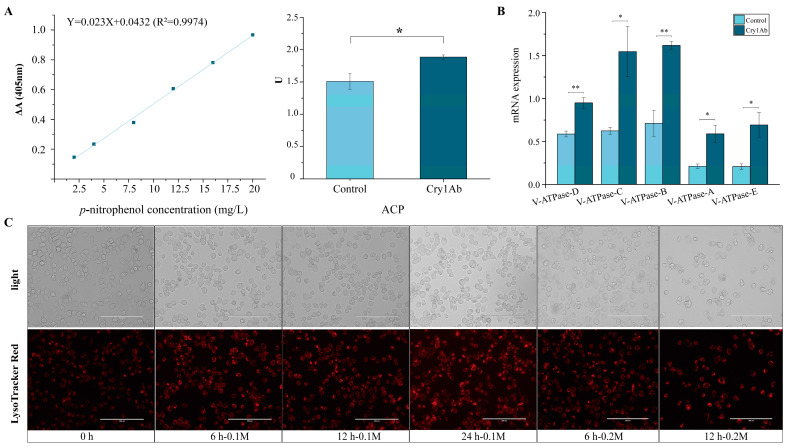
Effects of Cry1Ab exposure on lysosome activity in *S. litura* larvae. (**A**) The Acid Phosphatase activity and (**B**) The relative mRNA expression of V-ATPase genes after a 48 h sublethal Cry1Ab exposure in midgut. (**C**) Labeling for lysosomes (red). The asterisk on the bars indicates significantly different means (*: 0.01 ≤ *p* ≤ 0.05; **: *p* ≤ 0.01) performed by *t*-test.

Lysosomal ACP activity in the midguts of experimental insects increased after exposure to Cry1Ab for 48 h (Figure 4A). The mRNA expression of the V-ATPase genes was increased in the Cry1Ab-treated midguts (Figure 4B). Surprisingly, acidification visualized by LysoTracker Red staining demonstrated that the lysosome was highly sensitive to the Cry1Ab treatment (Figure 4C).

## 4. Discussion

An almost 30-year history of Bt use supports the biosafety of commercial Bt-derived products and demonstrates that it is an important control agent against insect pests. Cry1Ab enables the prospective control of various agricultural pests, such as corn earworms and *S. frugiperda*, as previously reported [29,30,31,32]. In this study, young first–fourth instars *S. litura* larvae had significant mortality rates when fed transgenic Bt DBN9936 maize expressing Cry1Ab toxin. While fifth instar larvae could mostly survive on the Cry1Ab plant, it had sublethal effects of inhibiting weight and development, as observed in *H. armigera*, *Leptinotarsa decemlineata*, *Trichogramma chilonis*, and *S. frugiperda* [33,34,35,36]. Hence, it is indicated that when planting a transgenic crop, for example, the DBN9936 corn, the population dynamics of the pests associated with crop development need to be investigated.

The accumulation and transport of Cry1Ab in *S. litura* larvae indicate that its regulation was achieved through the sequestration of the toxins from the lumen of the digestive system to excretion in the feces or through transport in a gut-to-hemolymph direction, where it rapidly accumulated in the cuticle and was then removed by molting. Generally, excretion is an efficient way to dispose of metabolized wastes and noxious materials in insects [37,38,39]. Raps et al. (2001) studied the Cry1Ab concentration in the feces of the herbivore *Spodoptera littoralis* (Boisduval) (Lepidoptera: Noctuidae) after feeding on Bt maize leaves [40]. Most research on the insect detoxification of xenobiotics or insecticides and the excretion of the resulting metabolites is based on Phase I (cytochrome P450s, CYP450; carboxyl/cholinesterases, CCEs), Phase II (UDP-glycosyl-transferases, UGTs; glutathione S-transferases, GSTs), and Phase III (ABC transporters) detoxification enzymes [41,42]. This is in accordance with our earlier studies showing that the gene expression levels of detoxification enzymes were mostly enhanced after exposure to sublethal Cry1Ablevels [27].

However, the increased Cry1Ab concentration was noted in the cast skin instead of the new cuticle, suggesting that molting is a new mechanism for Cry1Ab elimination in *S. litura*. Raessler et al. (2005) presented molting as a possible means of detoxification for woodlice under metal exposure [43]. Subsequently, transcriptomic analysis revealed that Cry toxin influences the formation of new cuticles during larvae molting [44,45]. In most cases, it is unknown how the Cry protein traverses the gut epithelium, enters the hemolymph, and becomes concentrated in the cuticle since the transport pathway of Bt toxin when ingested orally, has seldom been explored in insects. Interestingly, Bt toxins were detected in red palm weevil and *H. armigera* hemolymph when exposed to a sublethal dose of Bt, suggesting that Bt can bypass the gut to reach the hemolymph [46,47]. In *Agrotis ipsilon* (Hufnagel), phagocytosing hemocytes, intracellular *B. thuringiensis*, and attached bacteria were observed in a transmission electron micrograph [48]. In insect midgut epithelia, the molecular mechanisms underlying the transport of large protein molecules (>1 KDa) transport through the basolateral membrane remain poorly understood. Based on these results, we proposed that the interaction of the Cry toxin with the receptors on the apical membrane of the epithelia would trigger the inward budding of the membrane, creating a vesicle containing the imported Cry1Ab. This hypothesis is based on that created by Denecke et al. (2018) [49].

An analysis of the precise ultrastructure of the midgut epithelial cells clearly elucidated that exposure to sublethal Cry1Ab levels results in severe structural alterations in the midguts of *S. litura* larvae. The outstanding features shown in this study are endoplasmic reticulum-enriched fractions with ribosome degradation and an increase in lysosome numbers, which are similar to those observed in *Anticarsia gemmatalis* (Hübner) and *Aedes albopictus* (Skuse) [50,51,52]. Moreover, corresponding changes in acid phosphatase activity, V-ATPase gene expression, and the LysoTracker staining level were also investigated to represent lysosome activity under Cry1Ab stress. Collectively, our results indicate that exposure to sublethal Cry1Ab levels potentially increases lysosomal acidification. Based on the lysosome functions in cellular signaling, endocytotic, and macroautophagy pathways, lysosomal acidification activation raises the possibility that lysosomes are responsible for Cry1Ab transport and sequestration. However, the mechanisms present in *S. litura* larvae for the efflux or detoxification of Cry1Ab are not currently understood.

## 5. Conclusions

In conclusion, our results indicate that *S. litura* larvae exposed to Cry1Ab were differentially concentrated in different tissues and under sublethal exposure, suggesting that larvae possess a subtly regulated strategy in a tissue-specific way. Results of ultrastructural changes and lysosomal activity measurement in the midgut showed the potential regulatory roles of lysosomes in the Cry1Ab transport and elimination processes.

## Figures and Tables

**Table 1:** List of primers used in the study.

Gene	Gene Accession Number	Primer Name	Primer Sequences (5′→3′)
Reference gene (RPL7A)	XM_022970499.1	F	CGCCCTTTGCCGTAAGAT
		R	TTGTTGCCGAGGACACCAC
V-ATPase-D	XM_022979936.1	F	CAATGCGAGACCCTGGAAGA
		R	CCAAGAAGGTCGAGAGAGGC
V-ATPase-C	XM_022970793.1	F	ACGAGTGAAGTGCTTGGGTA
		R	TGCTGTAGGAGATGAGCCAG
V-ATPase-B	XM_022971637.1	F	ATGAAATTGCCGCCCAGATC
		R	ATAGTGGGGTCATTGGCCAA
V-ATPase-A	XM_022969645.1	F	ACCAACAAGTTCACGTCTGC
		R	TTGGCTTGCAGTGGTTTCTC
V-ATPase-E	XM_022959471.1	F	CTGGCTGAAGTACCCAAGGA
		R	AGTCACTCTGGGCCTTTGAA

## Data Availability

The data presented in this study are available upon request from the corresponding author.

## References

[1] Bravo A., Gómez I., Porta H., García-Gómez B.I., Rodriguez-Almazan C., Pardo L., Soberón M. (2013). Evolution of *Bacillus thuringiensis* Cry toxins insecticidal activity. Microb. Biotechnol..

[2] Frankenhuyzen K.V. (2009). Insecticidal activity of *Bacillus thuringiensis* crystal proteins. J. Invertebr. Pathol..

[3] Koch M.S., Ward J.M., Levine S.L., Baum J.A., Vicini J.L., Hammond B.G. (2015). The food and environmental safety of Bt crops. Front. Plant Sci..

[4] Romeis J., Meissle M., Raybould A., Hellmich R.L. (2009). Impact of Insect-Resistant Transgenic Crops on Aboveground Non-Target Arthropods.

[5] Martineau B. (2001). First Fruit: The Creation of the Flavr Savr Tomato and the bIrth of Biotech Foods.

[6] Li Y., Peng Y., Hallerman E.M., Wu K. (2014). Biosafety management and commercial use of genetically modified crops in China. Plant Cell Rep..

[7] Sun G.Q., Zhang D.L., Zhang R. (2016). Bt protein expression in the transgenic insect-resistant cotton in China. Sci. Bull..

[8] Li Y., Hallerman E.M., Liu Q., Wu K., Peng Y. (2016). The development and status of Bt rice in China. Plant Biotechnol. J..

[9] Li G., Ji T., Zhao S., Feng H., Wu K. (2022). High-Dose Assessment of Transgenic Insect-Resistant Maize Events against Major Lepidopteran Pests in China. Plants.

[10] Yang X., Zhao S., Liu B., Gao Y., Hu C., Li W., Yang Y., Li G., Wang L., Yang X. (2022). Bt maize can provide non-chemical pest control and enhance food safety in China. Plant Biotechnol. J..

[11] Yang F., Zheng K.L., Yao Y. (2024). China’s regulatory change toward genome-edited crops. Trends Biotechnol..

[12] Liu F., Xu Z., Zhu Y.C., Huang F., Wang Y., Li H., Li H., Gao C., Zhou W., Shen J. (2010). Evidence of field evolved resistance to Cry1Ac expressing Bt cotton in Helicoverpa armigera in northern China. Pest Manag. Sci..

[13] Dangal V., Huang F. (2015). Fitness costs of Cry1F resistance in two populations of fall armyworm, *Spodoptera frugiperda* (J.E. Smith), collected from Puerto Rico and Florida. J. Invertebr. Pathol..

[14] Bagla P. (2010). Hardy cotton-munching pests are latest blow to GM crops. Science.

[15] Dhurua S., Gujar G.T. (2011). Field-evolved resistance to Bt toxin Cry1Ac in the pink bollworm, *Pectinophora gossypiella* (Saunders) (Lepidoptera: Gelechiidae), from India. Pest Manag. Sci..

[16] Ferré J., Real M.D., Van Rie J., Jansens S., Peferoen M. (1991). Resistance to the Bacillus thuringiensis bioinsecticide in a field population of *Plutella xylostella* is due to a change in a midgut membrane receptor. Proc. Natl. Acad. Sci. USA.

[17] Reay-Jones FP F., Bilbo T.R., Reisig D.D. (2020). Decline in sublethal effects of Bt corn on *Corn Earworm* (Lepidoptera: Noctuidae) linked to increasing levels of resistance. J. Econ. Entomol..

[18] Pezzini D., Taylor K.L., Reisig D.D., Fritz M.L. (2024). Cross-pollination in seed-blended refuge and selection for Vip3A resistance in a Lepidopteran pest as detected by genomic monitoring. Proc. Natl. Acad. Sci. USA.

[19] Gaétan M., Éric B. (2001). Developmental Polymorphism: A major factor for understanding sublethal effects of *Bacillus Thuringiensis*. Entomol. Exp. Appl..

[20] Sedaratian A., Fathipour Y., Talaei-Hassanloui R., Jurat-Fuentes J.L. (2013). Fitness costs of sublethal exposure to Bacillus thuringiensis in Helicoverpa armigera: A carryover study on offspring. J. Appl. Entomol..

[21] Elkins B.H., Portilla M., Allen K.C., Little N.S., Mullen R.M., Paulk R.T., Read Q.D. (2024). Sublethal effects of a commercial Bt product and Bt cotton flowers on the bollworm (*Helicoverpa zea*) with impacts to predation from a lady beetle (*Hippodamia convergens*). PLoS ONE.

[22] Chauhan V.K., Dhania N.K., Chaitanya R.K., Senthilkumaran B., Dutta-Gupta A. (2017). Larval mid-gut responses to sub-lethal dose of Cry toxin in Lepidopteran pest *Achaea janata*. Front. Physiol..

[23] Hernández-Martínez P., Gomis-Cebolla J., Ferré J., Escriche B. (2017). Changes in gene expression and apoptotic response in *Spodoptera exigua* larvae exposed to sublethal concentrations of Vip3 insecticidal proteins. Sci. Rep..

[24] Chauhan V.K., Dhania N.K., Lokya V., Bhuvanachandra B., Padmasree K., Dutta-Gupta A. (2021). Midgut aminopeptidase N. expression profile in *Castor semilooper* (*Achaea janata*) during sublethal Cry toxin exposure. J. Biosci..

[25] Van Munster M., Prefontaine G., Meunier L., Elias M., Mazza A., Brousseau R., Masson L. (2007). Altered gene expression in *Choristoneura fumiferana* and *Manduca sexta* in response to sublethal intoxication by *Bacillus thuringiensis* Cry1Ab toxin. Insect Mol. Biol..

[26] Cheng T., Wu J., Wu Y., Chilukuri R.V., Huang L., Yamamoto K., Feng L.I., Li W., Chen Z., Guo H. (2017). Genomic adaptation to polyphagy and insecticides in a major East Asian noctuid pest. Nat. Ecol. Evol..

[27] Xu Y.J., Zhang Y.N., Hao S.P., Wang Y.J., Yang X.X., Shen Y.Q., Su Q., Xiao Y.D., Liu J.Q., Li W.S. (2024). Proteotranscriptomic analyses of the midgut and Malpighian tubules after a sublethal concentration of Cry1Ab exposure on *Spodoptera litura*. Pest Manag. Sci..

[28] Shu B., Zhang J., Cui G., Sun R., Sethuraman V., Yi X., Zhong G. (2018). Evaluation of reference genes for real-time quantitative PCR analysis in larvae of *Spodoptera litura* exposed to azadirachtin stress conditions. Front. Physiol..

[29] Reisig D.D., Reay-Jones FP F. (2015). Inhibition of *Helicoverpa zea* (Lepidoptera: Noctuidae) Growth by Transgenic Corn Expressing Bt Toxins and Development of Resistance to Cry1Ab. Environ. Entomol..

[30] Bilbo T.R., Reay-Jones F.P., Reisig D.D., Musser F.R., Greene J.K. (2018). Effects of Bt corn on the development and fecundity of Corn Earworm (Lepidoptera: Noctuidae). J. Econ. Entomol..

[31] Lone S.A., Malik A., Padaria J.C. (2017). Selection and characterization of *Bacillus thuringiensis* strains from northwestern Himalayas toxic against *Helicoverpa armigera*. Microbiologyopen.

[32] Botha A.S., Erasmus A., du Plessis H., Van den Berg J. (2019). Efficacy of Bt Maize for Control of *Spodoptera frugiperda* (Lepidoptera: Noctuidae) in South Africa. J. Econ. Entomol..

[33] Costa S.D., Barbercheck M.E., Kennedy G.G. (2000). Sublethal acute and chronic exposure of colorado potato beetle (Coleoptera: Chrysomelidae) to the δ-Endotoxin of *Bacillus thuringiensis*. J. Econ. Entomol..

[34] Amichot M., Curty C., Benguettat-Magliano O. (2016). Side effects of *Bacillus thuringiensis* var. kurstaki on the hymenopterous parasitic wasp *Trichogramma chilonis*. Environ. Sci. Pollut. Res..

[35] Tavares C.S., Santos-Amaya O.F., Oliveira E.E., Paula-Moraes S.V., Pereira E.J.G. (2021). Facing Bt toxins growing up: Developmental changes of susceptibility to Bt corn hybrids in fall armyworm populations and the implications for resistance management. Crop Prot..

[36] Bauce E., Kumbasli M., Van Frankenhuyzen K., Carisey N. (2006). Interactions Among White Spruce Tannins, *Bacillus thuringiensis* subsp. *kurstaki*, and Spruce Budworm (Lepidoptera: Tortricidae), on larval survival, growth, and Development. J. Econ. Entomol..

[37] Nishida R. (2002). Sequestration of defensive substances from plants by Lepidoptera. Annu. Rev. Entomol..

[38] Van Broekhoven S., Gutierrez J.M., De Rijk T.C., De Nijs W.C.M., Van Loon J.J.A. (2017). Degradation and excretion of the Fusarium toxin deoxynivalenol by an edible insect, the Yellow mealworm (*Tenebrio molitor* L.). World Mycotoxin J..

[39] Hagele B.F., Rowell-Rahier M. (1999). Dietary mixing in three generalist herbivores: Nutrient complementation or toxin dilution?. Oecologia.

[40] Raps A., Kehr J., Gugerli P., Moar W.J., Bigler F., Hilbeck A. (2001). Immunological analysis of phloem sap of *Bacillus thuringiensis* corn and of the nontarget herbivore *Rhopalosiphum padi* (Homoptera: Aphididae) for the presence of Cry1Ab. Mol. Ecol..

[41] Kshatriya K., Gershenzon J. (2024). Disarming the defenses: Insect detoxification of plant defense-related specialized metabolites. Curr. Opin. Plant Biol..

[42] Boeckler G.A., Paetz C., Feibicke P., Gershenzon J., Unsicker S.B. (2016). Metabolism of poplar salicinoids by the generalist herbivore *Lymantria dispar* (Lepidoptera). Insect Biochem. Mol. Biol..

[43] Raessler M., Rothe J., Hilke I. (2005). Accurate determination of Cd, Cr, Cu and Ni in woodlice and their skins--is moulting a means of detoxification?. Sci. Total Environ..

[44] Wang J., Peng Y., Xiao K., Wei B., Hu J., Wang Z., Song Q., Zhou X. (2017). Transcriptomic response of wolf spider, Pardosa pseudoannulata, to transgenic rice expressing *Bacillus thuringiensis* Cry1Ab protein. BMC Biotechnol..

[45] Pezenti L.F., Sosa-Gómez D.R., de Souza R.F., Vilas-Boas L.A., Gonçalves K.B., da Silva C.R.M., Vilas-Bôas G.T., Baranoski A., Mantovani M.S., da Rosa R. (2021). Transcriptional profiling analysis of susceptible and resistant strains of *Anticarsia gemmatalis* and their response to *Bacillus thuringiensis*. Genomics.

[46] Zhao Z., Li Y., Xiao Y., Ali A., Dhiloo K.H., Chen W., Wu K. (2016). Distribution and metabolism of Bt-Cry1Ac toxin in tissues and organs of the cotton bollworm, *Helicoverpa armigera*. Toxins.

[47] Manachini B., Arizza V., Parrinello D., Parrinello N. (2011). Hemocytes of *Rhynchophorus ferrugineus* (Olivier) (Coleoptera: Curculionidae) and their response to *Saccharomyces cerevisiae* and *Bacillus thuringiensis*. J. Invertebr. Pathol..

[48] El-Aziz N.M., Awad H.H. (2010). Changes in the haemocytes of *Agrotis ipsilon* larvae (Lepidoptera: Noctuidae) in relation to dimilin and *Bacillus thuringiensis* infections. Micron.

[49] Denecke S., Swevers L., Douris V., Vontas J. (2018). How do oral insecticidal compounds cross the insect midgut epithelium?. Insect Biochem. Mol. Biol..

[50] Castro B.M.D.C.E., Martinez L.C., Barbosa S.G., Serrão J.E., Wilcken C.F., Soares M.A., da Silva A.A., de Carvalho A.G., Zanuncio J.C. (2019). Toxicity and cytopathology mediated by *Bacillus thuringiensis* in the midgut of *Anticarsia gemmatalis* (Lepidoptera: Noctuidae). Sci. Rep..

[51] Silva V.C., Pinheiro N.L., Scherer P.O., Falcão S.S., Ribeiro V.R., Mendes R.M.M., Chagas R., Cardozo-De-Almeida M., Dos Santos-Mallet J.R. (2008). Histology and ultrastructure of *Aedes albopictus* larval midgut infected with *Bacillus thuringiensis* var. israelensis. Microsc. Res. Tech..

[52] Daquila B.V., Scudeler E.L., Dossi F.C.A., Moreira D.R., Pamphile J.A., Conte H. (2019). Action of *Bacillus thuringiensis* (Bacillales: Bacillaceae) in the midgut of the sugarcane borer Diatraea saccharalis (Fabricius, 1794) (Lepidoptera: Crambidae). Ecotoxicol. Environ. Saf..

